# Single center experience with surgically implanted Melody and Sapien 3 valves in the mitral position in young children

**DOI:** 10.3389/fcvm.2025.1584134

**Published:** 2025-06-13

**Authors:** Hüseyin Sicim, Mohamad Alaeddine, Daniel A. Velez

**Affiliations:** Department of Cardiac Surgery, Phoenix Children’s Hospital, Phoenix, AZ, United States

**Keywords:** melody, Sapien 3, mitral valve replacement, surgical, children

## Abstract

**Objectives:**

Mitral valve replacement in young children is a challenging situation due to limited options. In this study, we aimed to present the results of our patients who underwent mitral valve replacement using Melody (Medtronic Inc) and Sapien 3 (Edwards Inc) valves.

**Methods:**

We performed a total of 18 mitral valve replacements at Phoenix Children's between 2017 and 2024. These include 12 Melody and 6 Sapien valves. The median patient age was 42.17 ± 38.9 (1.9–127.2) months and weight was 13.83 ± 9.38 (3.9–43.4) kg, respectively. The indications for implantation were mitral stenosis with or without regurgitation, following an atrioventricular septal defect (AVSD) repair, mitral valve dysplasia, and Shone's complex. Once positioned the valves were expanded using a balloon catheter to achieve the best diameter (16–30 mm).

**Results:**

The postoperative mean mitral gradient was 6 ± 2.91 (2–12) mmHg. A permanent pacemaker was implanted in one patient after Sapien 3 valve replacement. One patient who received a Sapien valve had to be re-intervened for tamponade in the early post operative period. Mild LVOT obstruction was observed in one patient who underwent Melody valve replacement. One Melody valve was dilated after one year after implantation. At 24 months after implantation, Kaplan–Meier analysis indicated that 80% of Melody valves and 83% of Sapien valves would be expected to be free from reoperation (Log-rank *p* = 0,56).The hospital stay was 18.72 ± 3.67 days, and no mortality was observed.

**Conclusions:**

Melody and Sapien valves contribute to the prognosis of patients as it shortens the operation time, is easily applicable, and provides opportunities such as postoperative redilation. The successful results we obtained show that both valves can be used reliably and effectively and can be an alternative to conventional methods.

## Introduction

Mitral valve surgeries in young children presents a unique set of challenges due to the complexity of congenital heart defects and the limited options for valve replacement. Traditional surgical approaches often involve prosthetic valves that may not be suitable for the small anatomies and growth patterns seen in pediatric patients. Recent advancements have led to the exploration of stent-based valves, such as the Melody (Medtronic Inc., Minneapolis, USA) and Sapien 3 (Edwards Lifesciences Inc., California, USA) valves, which offer a promising alternative for addressing mitral valve dysfunction in this vulnerable population.

The Melody valve, originally designed for pulmonary valve replacement, has been repurposed for use in the mitral position, enabling physicians to leverage its expandable design to accommodate the anatomical constraints of young children (1–3). Similarly, the Sapien 3 valve, with its established efficacy in transcatheter aortic valve replacement, has also been evaluated for mitral valve replacement, offering the potential for less invasive approaches and improved postoperative outcomes (4–6).

This study aims to present our center's experience with mitral valve replacement in young children using the Melody and Sapien 3 valves. By analyzing clinical outcomes of 18 patients, we evaluated the efficacy and safety of these innovative approaches, contributing to the growing body of evidence that supports their use as reliable alternatives to traditional surgical methods.

## Methods

### Study design and population

This retrospective study was conducted at Phoenix Children's Heart Center between 2017 and 2024. Institutional Ethics committee approval was obtained from Review Board (IRB-21-235), and written informed consent was obtained from each patient. We identified patients who underwent mitral valve replacement using either Melody (Medtronic Inc) or Sapien 3 (Edwards Lifesciences Inc) valves. A total of 18 procedures were performed during this period, comprising 12 Melody valves and 6 Sapien 3 valves. Inclusion criteria involved patients under the age of 18 years who were diagnosed with conditions requiring MVR, including mitral stenosis, mitral regurgitation, atrioventricular septal defect (AVSD) repair, congenital mitral valve dysplasia, and Shone's complex. Patients were analyzed in terms of their fundamental diagnoses, age, demographic characteristics, postoperative complications, durability of stent-based valve, incidence of reoperation, other morbidities, and mortality.

### Preoperative assessment

All patients underwent comprehensive preoperative evaluations, including echocardiography to assess the severity of mitral valve pathology, cardiac anatomy, and hemodynamic status. Additional imaging studies, such as MRI or CT scans, were performed when indicated to provide detailed anatomical information. A multidisciplinary team, including pediatric cardiologists, cardiac surgeons, and anesthesiologists, participated in preoperative planning to determine the most appropriate surgical approach and valve selection for each patient.

### Surgical procedure

**Figure 1:**
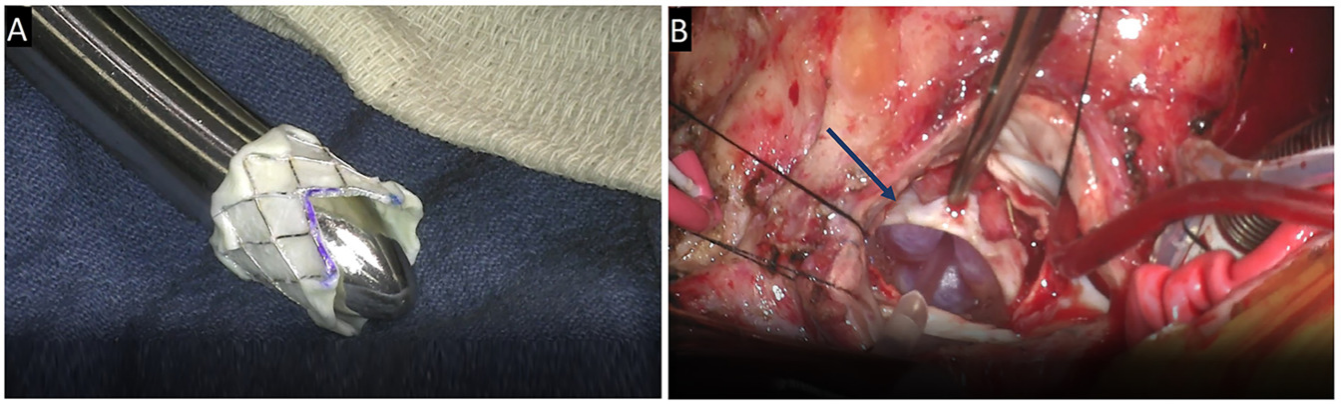
**(A)** Modified valve by cutting a V shape of the stent and **(B)** saline test is performed to ensure the valve's competency.

All surgical procedures were performed under general anesthesia using standard median sternotomy. Given the small size of our patients, we modify the Melody valves by cutting a V shape of the stent (Figure 1A). The cutting edges are oversewn to prevent de-gloving of the mounted valve. This approach can be adjusted based on our confidence in accommodating the ventricular component of the valve without inducing left ventricular outflow tract obstruction (LVOTO).

At this juncture, cardioplegia is administered. Depending on the dilation of the left atrium, we have successfully utilized both trans-septal and left atriotomy approaches for implantation. We aim to preserve the subvalvular apparatus whenever feasible. We routinely saw a ring of bovine pericardium to the annulus of the mitral valve. The Melody valve is crimped over the dilator using circumferential finger pressure and inserted into the bovine ring/mitral annulus. Afterward, pre-placed mitral annular sutures are passed through and tied to the sewing ring.

A partial dilatation of the valve may be conducted prior to securing the sutures, if necessary. Once tied, the valve is gradually dilated, based on the anticipated valve size and the dimensions of the available annulus. A saline test is performed to ensure the valve's competency (Figure 1B). The interatrial septum may be augmented using a fenestrated bovine pericardium patch to enhance left atrial volume. A brief examination of both pulmonary veins ensures unobstructed flow into the valve.

### Statistical analysis

The Kaplan–Meier product-limit method was utilized to evaluate the freedom from MV reoperation and cumulative incidence of MV reoperation while accounting for censoring, with 95% confidence intervals (CIs) calculated using Greenwood's formula. The median postoperative gradient and amount of regurgitation were compared by the nonparametric Wilcoxon signed-rank test. The valve sizes were compared using the paired *t* test. All statistical analyses were conducted using SAS 9.4 (SAS Institute), with a two-tailed *P* value of less than 0.05 deemed statistically significant.

## Results

A total of 18 stent-based mitral valve replacements were performed at our institution between 2017 and 2024. 12 patients received the Melody valve, and 6 patients received the Sapien valve. The demographic characteristics, fundamental diagnosis, postoperative complications and follow up of the patients are detailed in Tables 1, 2. The median age of the patients at the time of surgery was 42.17 ± 38.9 months, with an age range from 1.9–127.2 months. The median weight was recorded at 13.83 ± 9.38 kg, with a range of 3.9 kg–43.4 kg.

**Table 1:** Summary of the patients who underwent surgical placed Melody valves.

No.	Fundamental Diagnosis	Valve	Age at Surgery (months)	Weight at Surgery (kg)	Preoperative mean gradient (mmHg)	Preoperative mitral regurgitation	Postoperative mean grafient (mmHg)	Postoperative mitral regurgitation	Follow up (years)	Length of hospital stay (day)	Postoperative Complication
1	Congenital mitral valve stenosis and dysplasia	Melody	1.9	3.9	11	Severe	3	Trivial	1	22	No
2	Congenital mitral valve dysplasia	Melody	5.9	7	19	Mild	7	Trivial	3	7	No
3	Complete AV canal defect	Melody	5.7	6.93	10	Severe	8	Mild	2	48	Mild LVOT obstruction
4	Complete AV canal defect	Melody	5.7	6.12	17	Severe	5	Trivial	4	5	No
5	Aortic arch hypoplasia, mitral regurgitation	Melody	24.5	8	12	Severe	3	Mild	2	6	Bilateral pleural effusions
6	Aortic arch hypoplasia, mitral stenosis	Melody	24.5	8.57	14	Modeate	6	Trace	7	21	No
7	Shone's syndrome	Melody	14.1	11.4	12	Severe	5	Mild	5	19	Benign arrhytmia
8	Aortic arch hypoplasia, mitral stenosis	Melody	38.9	11.37	21	Severe	12	Mild	4	41	No
9	Mitral regurgitation, parachute mitral valve	Melody	42.4	14.3	19	Severe	8	Mild	5	46	ARDS
10	Mitral regurgitation, Rheumatic	Melody	38.9	11.37	5	Severe	2	Mild	5	17	No
11	Mitral regurgitation and stenosis	Melody	127.2	22.6	15	Moderate	6	Trivial	2	13	No
12	Complete AV canal defect	Melody	69.1	23.25	13	Severe	5	Trivial	2	24	Mild LV aneurysm (No intervention required)

**Table 2:** Summary of the patients who underwent surgical placed Sapien 3 valves.

No.	Fundamental Diagnosis	Valve	Age at Surgery (months)	Weight at Surgery (kg)	Preoperative mean gradient (mmHg)	Preoperative mitral regurgitation	Postoperative mean gradient (mmHg)	Postoperative mitral regurgitation	Follow up (years)	Length of hospital stay (day)	Postoperative Complication
1	Mitral regurgitatian and stenosis	Sapien	20.5	12	18	Severe	5	Trivial	4	6	Pericarditis (Small pericardial effusion)
2	Severe mitral stenosis	Sapien	25.4	10.39	16	Mild	8	Trivial	3	8	No
3	Common atrium and mitral stenosis	Sapien	50	11.5	11	Severe	5	Trace	1	7	No
4	Complete AV canal defect	Sapien	39.2	12.9	2	Severe	2	Trace	4	10	Cardiac tamponade
5	Mitral regurgitation, Rheumatic	Sapien	118.7	43.4	15	Moderate	3	Mild	3	22	Pleural effusion
6	Mitral regurgitation, parachute mitral valve	Sapien	106.6	24	13	Severe	3	Trivial	3	15	Complete AV block (PM placed)

The primary indications for surgery included mitral stenosis (*n* = 5), mitral regurgitation (*n* = 5), both mitral stenosis and regurgitation (*n* = 2), atrioventricular septal defect (AVSD) repair (*n* = 4), congenital mitral valve dysplasia (*n* = 1), and Shone's complex (*n* = 1).

**Figure 2:**
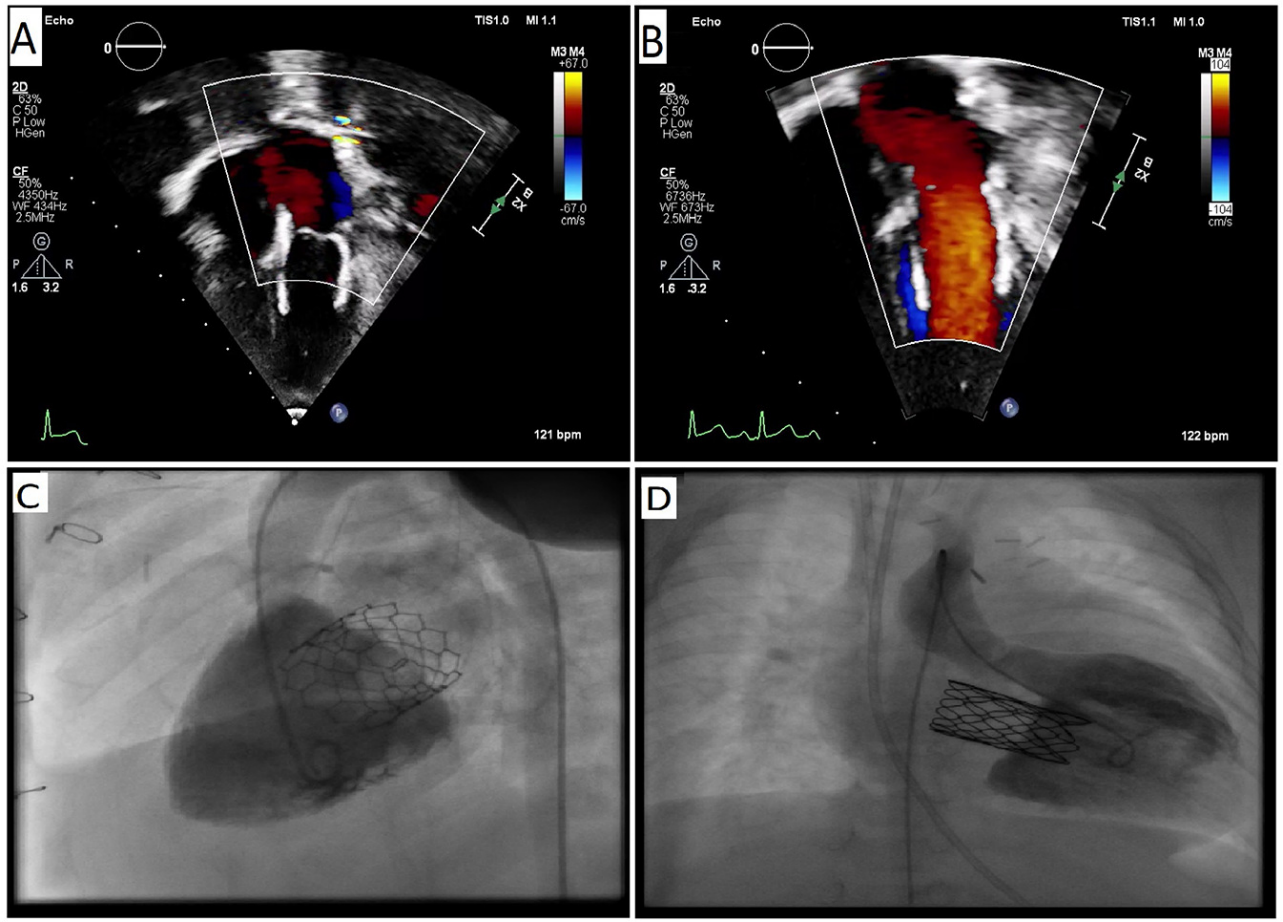
**(A)** Two-dimensional transthoracic echocardiography, **(B)** color doppler image of the stent-based valve in mitral position, **(C)** left ventriculogram showing no mitral regurgitation in Sapien 3 and **(D)** Melody implanted patients.

The mean postoperative mitral gradient was 6 ± 2.90 mmHg, with a range of 2–12 mmHg (Table 1). Postoperative transthoracic echocardiographic imaging revealed trivial or mild regurgitation in all patients (Figure 2). This outcome indicates satisfactory valve function and hemodynamic performance in most patients. The hospital stay was 18.72 ± 13.67 days, and no mortality was observed in any patient.

**Figure 3:**
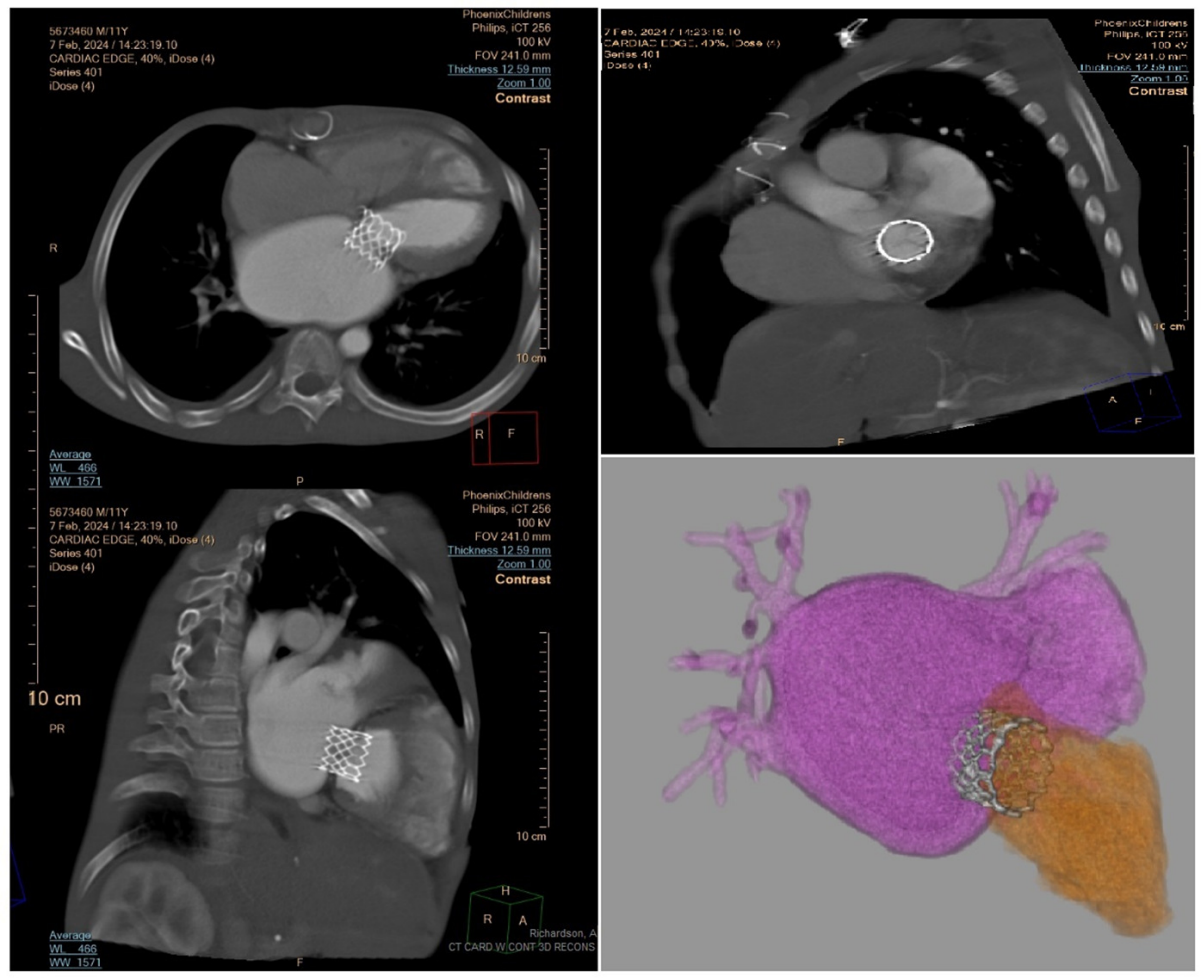
Computed tomography multiplanar reformation, cross-sectional and 3-dimensional models of implanted stent-based mitral valve at postoperative follow up.

A total of four significant complications were noted among the patients. One patient who received a Sapien 3 valve required the placement of a temporary pacemaker due to bradycardia occurring in the early postoperative phase. This event underscores the importance of monitoring cardiac rhythms closely in this patient population. One patient with a Melody valve experienced prolonged hospitalization due to acute respiratory distress syndrome (ARDS). This patient required supportive care, including mechanical ventilation, highlighting the potential respiratory risks associated with cardiac surgery in infants and young children. A case of cardiac tamponade was identified in a patient who underwent Sapien 3 valve replacement, necessitating urgent intervention. The patient was successfully treated through pericardiocentesis. Mild left ventricular outflow tract (LVOT) obstruction was observed in one patient post-Melody valve replacement. The condition was closely monitored, and the patient remained asymptomatic at the ongoing follow-up. In case of indications related to the patient's comorbid factors or directly to the mitral valve, valve competence was assessed by evaluation in the catheter laboratory (Figure 2). Again, in the postoperative period, in case of relevant indication, computed tomography and, if necessary, 3D configuration contributed to the detailed evaluation of the valve position (Figure 3).

No patients exhibited pulmonary venous obstruction, which may be a significant concern in pediatric mitral valve replacement. The absence of this complication indicates a favorable safety profile for both valve types. At one-year follow-up, one patient with a Melody valve underwent a successful balloon dilation procedure, reflecting the potential for device adjustments as the patient grows. This ability for postoperative redilation is particularly beneficial in the pediatric population, potentially reducing the frequency of reoperations.

**Figure 4:**
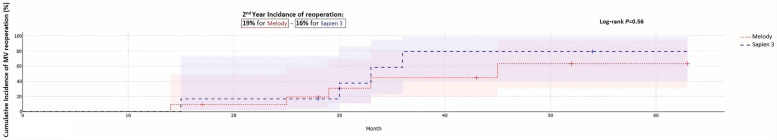
Kaplan-Meier curves showing cumulative incidence of mitral valve reoperation.

**Figure 5:**
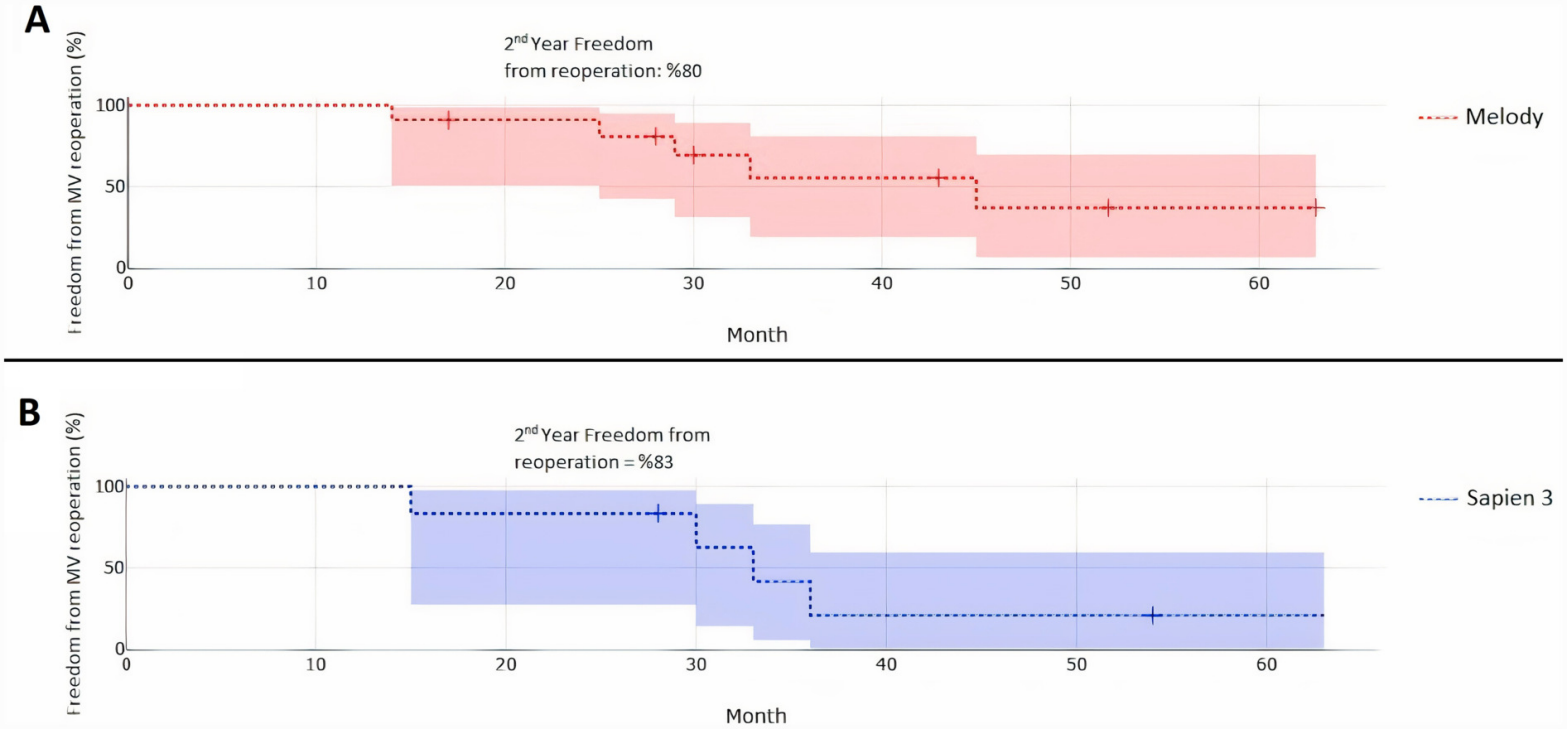
**(A)** Kaplan-Meier curves showing the freedom from mitral reoperations in melody patients and **(B)** the freedom from mitral reoperations in Sapien 3 patients.

At 24 months after implantation, Kaplan–Meier analysis showed that the cumulative incidence of reoperation at 24 months after placement was 19% in Melody patients and 16% in Sapien 3 patients (*p* = 0.56) (Figure 4). And Kaplan–Meier analysis indicated that 80% for Melody (Figure 5A) and 83% for Sapien 3 (Figure 5B) would be expected to be free from reoperation at 24 months after surgery (Log-rank *p* = 0.56). Throughout the study period, there were no recorded deaths among the cohort. All patients were discharged in stable condition, highlighting the overall success of the procedures.

## Discussion

The results of our study highlight the evolving landscape of mitral valve replacement in young children, particularly using stent-based valves like the Melody and Sapien 3. Given the inherent complexities associated with congenital heart defects and the unique anatomical considerations in this patient demographic, the ability to leverage expandable valve technology offers a promising alternative to traditional surgical options (7, 8).

Our findings indicate that both the Melody and Sapien 3 valves are not only feasible but also effective for mitral valve replacement in infants and young children. The low mean postoperative mitral gradient of 5 mmHg and the absence of significant pulmonary venous obstruction across all patients underscore the favorable hemodynamic performance of these valves. Furthermore, the observed trivial to mild regurgitation in echocardiographic assessments suggests that both devices maintain competency postoperatively, which is crucial for the long-term success of valve replacements in this vulnerable population.

While the overall outcomes were promising, some complications were observed that warrant discussion. The need for permanent pacemaker placement in one patient post-Sapien 3 valve implantation highlights the importance of careful perioperative management and monitoring of cardiac rhythms. Similarly, the case of acute respiratory distress syndrome in a Melody valve patient point to the inherent risks associated with cardiac surgery in young children. Such respiratory complications necessitate thorough postoperative care and may influence recovery trajectories. Additionally, mild LVOT obstruction noted in one patient after Melody valve replacement raises considerations for valve modification and positioning and the preservation of subvalvular structures. Our strategy of retaining anterior and posterior chordal tissues appears beneficial; however, close monitoring remains essential to address potential obstructions that may arise from anatomical constraints.

The potential for postoperative redilation is a noteworthy advantage of stent-based valves, particularly in a growing pediatric population. Our experience with one patient who underwent successful balloon dilation after Melody valve implantation exemplifies the adaptability of these devices, allowing for growth without necessitating surgical reintervention. This adaptability could significantly alter the management of young patients with mitral valve disease, promoting better long-term outcomes and quality of life. As we move forward, further studies with larger cohorts and longer follow-up periods will be vital to validate our findings and explore the longevity of these valves. Continued investigation into optimal surgical techniques, valve sizing, and management of complications will enhance our understanding and application of stent-based valve technology.

Practitioners utilizing the stent-based valve generally adhere to established principles regarding its construction. It is crucial to optimize the valve's length without compromising the integrity of the stent. To maintain radial and longitudinal stability, we have chosen to resect stent struts, and we employ techniques that minimize ventricular profiling to mitigate the risk of LVOT obstruction.

As a traditional fashion, mechanical valves, known for their challenges in pediatric populations, have significant drawbacks, including high mortality rates and complications like thrombosis and permanent pacemaker requirements. Metras et al., in their study, reported that mechanical mitral valve replacement in young children, especially those under 2 years of age, causes high morbidity and mortality (9). In contrast, biological valves, while requiring no anticoagulation, are prone to faster degeneration, necessitating more frequent replacements (10, 11). Traditionally, biological valves do not necessitate long-term anticoagulation; however, the recommendations for the stent-based valve in the mitral position are evolving. Given our experience, we favor a cautious approach, initially recommending therapeutic dual antiaggregant for the first-year post-implantation, by aspirin and clopidogrel if echocardiographic evaluations remain favorable.

Another issue based on our center experience, creating a neo-sewing ring offers significant advantages. A prestitched sewing ring not only streamlines the suturing process but also effectively reduces the risk of paravalvular leaks, enhancing the overall stability of the implant. Maintaining the integrity of the subvalvular apparatus during mitral valve replacement has been linked to improved left ventricular function. Despite concerns regarding the interaction between the stent-based valve and small left ventricular cavities, our experience indicates that partial preservation of subvalvular structures is beneficial. This approach helps prevent systolic tilting of the valve into the LVOT, thus enhancing overall hemodynamic performance.

The ability to dilate the stent-based valve in response to growth is a critical feature of its design. Dranseika et al., in their study on the Melody valve, they have found that leaving a small fenestration in the atrial septum facilitates future interventions. In their cohort, significant reductions in transvalvular gradients were achieved through balloon dilatation without introducing new regurgitation or complications, highlighting the valve's adaptability (12). Emani et al., performed Melody valve implantation in aortic, mitral, pulmonary, or tricuspid positions. They advocated the following because of the satisfactory results they obtained compared to traditional methods; This technology may allow for a delay in reoperation in appropriately selected patients (13). Malik et al., performed the Melody valve on 5 patients, determined that the 24-month freedom from reoperation rate was over 60% and they recommend this method due to the positive results they obtained overall (14). Also in our literature review, Albacker et al. reported a case report in which they successfully applied Sapien 3 valve to a 75-year-old patient (15). Apart from this, there are limited patients in the literature who have undergone Sapien 3 implantation with the transcatheter method in the adult patient group (16, 17). In this respect, our stent-based series including Melody and Sapien 3 valve implantation in the mitral position in our study will make a significant contribution to science and has a unique value because of limited report in the literature.

Future design enhancements could mitigate many of the challenges encountered. The focus should be on optimizing the valve's length and attachment mechanisms to minimize surgical modifications and potential complications. Streamlining these aspects could significantly improve outcomes and reduce operative times.

Based on our findings, stent-based valves such as the Melody and Sapien 3 offer a promising alternative to traditional mitral valve replacement options in infants and young children, particularly due to their favorable hemodynamics, adaptability to somatic growth, and reduced need for early reoperation. We recommend their use in cases where valve repair is not feasible, with careful attention to valve positioning, preservation of subvalvular structures, and creation of a neo-sewing ring to optimize stability and reduce complications.

## Conclusion

Our findings advocate for the use of Melody and Sapien valves as effective options for mitral valve replacement in infants and young children. The ability of these valves to maintain effective hemodynamics, alongside their adaptability for growth, positions them as preferable alternatives to traditional prosthetic valves in this vulnerable demographic. Our study contributes to the emerging evidence base supporting the application of stent-based valve technology in the pediatric population, and further research is warranted to explore long-term outcomes and broader applications of these techniques.

## Data Availability

The original contributions presented in the study are included in the article/Supplementary Material, further inquiries can be directed to the corresponding author.

## References

[1] Patel ND Levi DS Cheatham JP Qureshi SA Shahanavaz S Zahn EM. Transcatheter pulmonary valve replacement: a review of current valve technologies. J Soc Cardiovasc Angiogr Interv. (2022) 1(6):100452. 10.1016/j.jscai.2022.100452 39132347 PMC11307711

[2] Freud LR Marx GR Marshall AC Tworetzky W Emani SM. Assessment of the Melody valve in the mitral position in young children by echocardiography. J Thorac Cardiovasc Surg. (2017) 153(1):153–160.e1. 10.1016/j.jtcvs.2016.07.017 27523403 PMC5864288

[3] Quiñonez LG Breitbart R Tworetsky W Lock JE Marshall AC Emani SM. Stented bovine jugular vein graft (Melody valve) for surgical mitral valve replacement in infants and children. J Thorac Cardiovasc Surg. (2014) 148(4):1443–9. 10.1016/j.jtcvs.2013.10.059 24332108

[4] Paradis JM Rodés-Cabau J. Transcatheter aortic valve replacement with the SAPIEN 3 valve: preparing the field for the final expansion. Cardiovasc Diagn Ther. (2017) 7(1):11–5. 10.21037/cdt.2016.11.04 28164008 PMC5253444

[5] Lamelas J Alnajar A. Early outcomes for surgical minimally invasive SAPIEN 3 transcatheter mitral valve replacement. Ann Thorac Surg. (2021) 112(2):494–500. 10.1016/j.athoracsur.2020.09.024 33144106

[6] Cabalka AK Asnes JD Balzer DT Cheatham JP Gillespie MJ Jones TK Transcatheter pulmonary valve replacement using the melody valve for treatment of dysfunctional surgical bioprostheses: a multicenter study. J Thorac Cardiovasc Surg. (2018) 155(4):1712–1724.e1. 10.1016/j.jtcvs.2017.10.143 29395214

[7] Choi PS Sleeper LA Lu M Upchurch P Baird C Emani SM. Revisiting prosthesis choice in mitral valve replacement in children: durable alternatives to traditional bioprostheses. J Thorac Cardiovasc Surg. (2021) 161:P213–225.E3. 10.1016/j.jtcvs.2020.04.173 32713632

[8] Overman DM Moga FX Stephens EH Dearani JA MacIver RH. Infant mitral valve replacement: current state of the art. Semin Thorac Cardiovasc Surg Pediatr Card Surg Annu. (2023) 26:75–80. 10.1053/j.pcsu.2023.01.001 36842801

[9] Metras A Seguela PE Roubertie F. Mechanical mitral valve replacement in children: an update. Transl Pediatr. (2019) 8(5):455–7. 10.21037/tp.2019.08.03 31993360 PMC6970109

[10] Marro M Kossar AP Xue Y Frasca A Levy RJ Ferrari G. Noncalcific mechanisms of bioprosthetic structural valve degeneration. J Am Heart Assoc. (2021) 10(3):e018921. 10.1161/JAHA.120.018921 33494616 PMC7955440

[11] Nitsche C Kammerlander AA Knechtelsdorfer K Kraiger JA Goliasch G Dona C Determinants of bioprosthetic aortic valve degeneration. J Am Coll Cardiol Img. (2020) 13(2_Part_1):345–53. 10.1016/j.jcmg.2019.01.027 30878425

[12] Dranseika V Pretre R Kretschmar O Dave H. Melody valve to replace the mitral valve in small children: lessons learned. Ann Pediatr Card. (2021) 14:35–41. 10.4103/apc.APC_74_20 PMC7918024 33679059

[13] Emani SM Piekarski BL Zurakowski D Baird CA Marshall AC Lock JE Concept of an expandable cardiac valve for surgical implantation in infants and children. J Thorac Cardiovasc Surg. (2016) 152(6):1514–23. 10.1016/j.jtcvs.2016.08.040 27692768

[14] Malik T Jaquiss RD Harirah O Davies RR Andersen N Leonard S The melody valve in small children undergoing first mitral valve replacement: better than mechanical? Ann Thorac Surg Short Rep. (2024) 2(3):359–63. 10.1016/j.atssr.2024.04.016 39790426 PMC11708157

[15] Albacker TB Bakir B Eldemerdash A Elshaer F Albacker H Alawami M Surgical mitral valve replacement using direct implantation of Sapien 3 valve in a patients with severe mitral annular calcification without adjunctive techniques, a case report. J Cardiothorac Surg. (2020) 15:42. 10.1186/s13019-020-1083-8 32093723 PMC7041179

[16] Kar B Nascimbene A Gregoric ID Patel M Loyalka P. Transcatheter mitral valve replacement with the Edwards Sapien 3 valve. Tex Heart Inst J. (2017) 44(4):269–73. 10.14503/THIJ-16-5823 28878582 PMC5577954

[17] Polomsky M Koulogiannis KP Kipperman RM Cohen BM Magovern CJ Slater JP Mitral valve replacement with Sapien 3 transcatheter valve in severe mitral annular calcification. Ann Thorac Surg. (2017) 103(1):e57–9. 10.1016/j.athoracsur.2016.06.105 28007276

